# Universal Newborn Screening for Congenital Cytomegalovirus Using Dried Blood Spot Specimens

**DOI:** 10.1001/jamanetworkopen.2025.54518

**Published:** 2026-01-29

**Authors:** Norma P. Tavakoli, Virginia Sack, Andrew S. Handel, Alyssa Giacinto, Melissa Pearce, Ifeyinwa Ojukwu, Charity McManaman, Marc St-Pierre, Lisa DiAntonio, Carlos Saavedra-Matiz, Christopher J. Brandon, Lequela Steen, Christine M. Salvatore, Sunil Sood, Julia A. Piwoz, Minnie John, Patricia DeLaMora, Sheila M. Nolan, Stephanie P. Ungar, Leonard B. Weiner, Danielle Daniels, Jennifer L. Nayak, Michael Quinn, Geoffrey A. Weinberg, Mark D. Hicar, Gitanjali Rebello, Gillian Taormina, Jency M. Daniel, Saul Hymes, Sharon Nachman, Sarah Bradley, Denise M. Kay, Michele Caggana

**Affiliations:** 1 Division of Genetics, Wadsworth Center, New York State Department of Health, Albany; 2 Department of Biomedical Sciences, State University of New York, Albany; 3 Department of Pediatrics, Renaissance School of Medicine at Stony Brook University, Stony Brook, New York; 4 Division of Pediatric Infectious Diseases, Weill Cornell Medicine, New York, New York; 5 Division of Infectious Diseases, Cohen Children’s Medical Center, Northwell, New Hyde Park, New York; 6 Division of Infectious Diseases, Department of Pediatrics, Albert Einstein College of Medicine, Children’s Hospital at Montefiore, Bronx, New York; 7 Pediatric Infectious Disease, New York Presbyterian Hospital, Brooklyn, New York; 8 Pediatric Infectious Diseases, New York Medical College, Valhalla, New York; 9 Department of Pediatrics, NYU (New York University) Grossman School of Medicine, New York, New York; 10 Department of Pediatrics, SUNY (State University of New York) Upstate Medical University, Syracuse; 11 Department of Pediatrics, University of Rochester School of Medicine and Dentistry, UR-Medicine Golisano Children’s Hospital, Rochester, New York; 12 Department of Pediatrics, Division of Infectious Diseases, Jacobs School of Medicine and Biomedical Sciences, Buffalo, New York; 13 Department of Pediatrics, Albany Medical College–Bernard & Millie Duker Children’s Hospital, Albany, New York

## Abstract

**Importance:**

Congenital cytomegalovirus (cCMV) is the most common nongenetic cause of sensorineural hearing loss in children and a cause of long-term neurological disabilities. In the absence of universal screening, cCMV disease in many newborns will go undiagnosed and untreated.

**Objective:**

To determine the feasibility, screen-positive rate, and parental acceptance of newborn screening for cCMV using dried blood spot (DBS) specimens.

**Design, Setting, and Participants:**

In this population-based diagnostic study, newborns whose DBS specimens were submitted to the New York State Newborn Screening Program from October 2, 2023, through September 30, 2024, for routine screening were also screened for CMV. Parents were given the option to opt out of receiving CMV results. Infectious disease specialists at designated medical centers performed follow-up of referred newborns. Of the 208 322 newborns whose specimens were submitted, 245 (0.1%) were opted out of the CMV screen by their parents; 22 families did so after CMV results were released.

**Exposure:**

DBS screening of newborns.

**Main Outcomes and Measures:**

CMV was detected in DBS specimens from newborns using a quantitative polymerase chain reaction analysis. Appropriate follow-up and treatment were provided to referred newborns.

**Results:**

Of the 208 099 newborns (48.6% female and 51.0% male; mean [SD] age at specimen collection, 3.5 [12.3] days [median, 1.0 days]) whose CMV results were reported, 529 had positive CMV screen results (0.3%). Following referral and initial diagnostic evaluation, 276 of the 529 newborns (52.2%) were diagnosed with cCMV (overall rate of 0.1%). Among the 276 newborns with cCMV, 68 (24.6%) had symptomatic cCMV disease, 197 (71.4%) had asymptomatic infection, and 11 (4.0%) had isolated sensorineural hearing loss. Additionally, 131 of the 529 referred newborns (24.8%) had likely acquired CMV postnatally, 17 (3.2%) had false-positive screen results, 43 (8.1%) had unknown CMV classification, and 62 (11.7%) were lost to follow-up or their parents declined follow-up. Of the 68 newborns with symptomatic cCMV disease, 48 (70.6%) were treated with antiviral medication.

**Conclusions and Relevance:**

In this diagnostic study, early identification of cCMV allowed newborns to be evaluated, which provided an opportunity for improved outcomes. Although the intent of the screening was to detect cCMV, a sizeable minority of cases identified had postnatally acquired CMV infections. Further studies that incorporate long-term data are needed to better understand the impact of cCMV identification among newborns with asymptomatic infection.

## Key Points

**Question:**

What is the feasibility and screen-positive rate of newborn dried blood spot screening for congenital cytomegalovirus (cCMV)?

**Findings:**

In this diagnostic study, 208 322 newborns were screened for cCMV, and screening results were reported for 208 099. CMV was detected in 529 newborns (1 in 393), of whom 276 (1 in 754) were diagnosed with cCMV, including 68 with symptomatic cCMV disease, 197 with asymptomatic cCMV infection, and 11 with isolated sensorineural hearing loss; another 131 (1 in 1589) had postnatally acquired CMV.

**Meaning:**

These findings suggest that newborn screening for cCMV is feasible but is hindered by factors including assay sensitivity and frequent identification of newborns with postnatally acquired CMV.

## Teaser

This diagnostic study describes the results of a 1-year pilot program in New York State to screen dried blood spots from newborns for congenital cytomegalovirus and evaluates the challenges and benefits of performing this screening.

## Introduction

Cytomegalovirus (CMV) is the most common cause of nonhereditary hearing loss in newborns. Congenital CMV (cCMV) prevalence is estimated to range from 0.48% in high-income countries to 1.42% in moderate- and low-income countries.^1^ Approximately 10% of newborns with cCMV exhibit symptoms, including sensorineural hearing loss (SNHL), microcephaly, jaundice, hepatosplenomegaly, and petechiae, and 40% to 50% of these newborns will have serious sequelae during childhood.^2,3^ Furthermore, imaging studies may exhibit intracranial calcifications, white matter lesions, ventriculomegaly, and polymicrogyria.^4,5^ Additionally, approximately 10% to 15% of newborns with asymptomatic cCMV develop sequelae later in life.^3,6,7^ The burden of CMV disease in newborns is therefore substantial, and efforts to prevent, diagnose, and treat CMV infection are of public health importance.

Early detection and antiviral treatment within the first months of life improve hearing and potentially neurodevelopmental outcomes among newborns with moderately to severely symptomatic cCMV disease^8,9,10^ or isolated SNHL.^11^ Targeted cCMV screening in newborns with failed universal newborn hearing screening can provide the opportunity for early intervention and treatment.^12^ Several states have mandated such hearing-targeted cCMV screening.^13^ However, asymptomatic newborns with late-onset hearing loss and premature newborns who do not undergo hearing screening prior to 3 weeks of age would not be identified through hearing-targeted screening.^14^ There is currently no method for determining which asymptomatic newborns will experience late-onset hearing deficits.

Interest in universal newborn screening (NBS) for cCMV has grown in recent years, as dried blood spot (DBS) screening for CMV has been shown to be feasible.^15,16,17^ Testing must be performed prior to 2 to 3 weeks of life because a positive result after this time could also be attributed to natal or postnatal infection.^18,19^ There are at least 3 NBS programs (NBSPs) in Canada and the US that are currently screening for cCMV.^20,21^ The Eunice Kennedy Shriver National Institute of Child Health and Human Development funded a 1-year pilot study in New York to screen DBS from newborns for cCMV and to better understand the challenges and benefits of performing NBS for CMV. Herein we describe the initial results of this pilot study.

## Methods

The New York State NBSP performed opt-out universal screening for cCMV from October 2, 2023, through September 30, 2024. This diagnostic study was approved by the New York State Department of Health Institutional Review Board, which granted a waiver of informed consent. The study followed the Strengthening the Reporting of Observational Studies in Epidemiology (STROBE) reporting guideline.

Screening was performed using a DBS quantitative polymerase chain reaction (PCR)–based assay. The New York State NBSP requires that for routine NBS, the DBS specimen be collected at 24 to 36 hours of age. For newborns in the neonatal intensive care unit (NICU), one specimen must be collected at admission, a second at 24 to 72 hours, and a third at 28 days of age or discharge. CMV testing was performed on all initial and repeat NBS specimens received, provided the sample was of suitable quality and sufficient volume. Additionally, repeat specimens received by the NBSP between October 1 and December 31, 2024, from newborns previously screened for cCMV were also tested. CMV screening results from newborns whose families opted out prior to results reporting (typically within 3 days of specimen receipt) were expunged and not reported. Educational materials were developed for clinicians at New York State birthing hospitals and those attending home births. An educational brochure for new parents was also developed (available in 17 languages) and described multiple methods for opting out.

### CMV DBS Assay

For CMV screening, nucleic acid was extracted from two 3.2-mm DBS punches using a commercially available DNA extraction reagent (Extracta DBS; Quantabio). Nucleic acid amplification and detection was achieved using a cCMV PCR reagent kit (NeoMDx; Revvity). Details of the assay and screening process are described in eMethods in Supplement 1.

### CMV Confirmatory Testing

Newborns with screen-positive results were referred to 1 of 11 designated referral centers in New York overseen by pediatric infectious diseases specialists (including S.S., C.M.S., J.A.P., M.J., P.D., S.M.N., S.P.U., L.B.W., D.D., J.L.N., M.Q., G.A.W., M.D.H., G.R., G.T., J.M.D., S.H., and S.N.) experienced in the evaluation and management of cCMV. Confirmatory CMV testing included urine PCR or culture; if these results were positive, newborns were categorized as having a confirmed CMV infection. One case was considered confirmed with results of saliva PCR. Newborns with a positive DBS screen result but preferably 2 negative confirmatory test results were considered to have a false-positive CMV screen result. Once alerted to a false-positive test, the NBSP retested both the DBS and blank spots on the NBS card (portions that did not include dried blood) from the original specimen. If a blank spot was positive for CMV, it indicated contamination of the NBS card. Newborns with confirmed CMV were determined to have confirmed cCMV if a positive DBS screen result was collected within the first 21 days of age. Clinicians also informed the NBSP of newborns who had a negative CMV DBS screen result but were clinically diagnosed with cCMV by urine PCR or culture results obtained prior to 21 days of age (false-negative CMV screen results). These newborns were often identified through institution-based universal screening in the NICU or routine diagnostic testing in symptomatic newborns.

### cCMV Clinical Evaluation

Newborns with confirmed cCMV underwent a diagnostic evaluation and clinical categorization guided by the consensus guidelines of Rawlinson et al.^18^ The recommended initial diagnostic evaluation for newborns with confirmed cCMV included a maternal and prenatal medical history, physical examination, laboratory testing (complete blood count with differential and measurement of alanine transaminase and total and direct bilirubin levels), brain ultrasonography, and audiological assessment. Ophthalmologic assessment was performed at the treating clinician’s discretion. Clinicians were also asked to report whether newborns with screen-positive results and symptomatic disease were clinically recognized with cCMV prior to receiving the positive DBS result.

### cCMV and non-cCMV Categorization

Newborns with confirmed cCMV were characterized by disease severity.^18^ Categories included asymptomatic cCMV infection (no abnormalities identified on diagnostic evaluation), asymptomatic cCMV infection with isolated SNHL (presence of SNHL without other abnormalities; referred to herein as isolated SNHL), or symptomatic cCMV disease (meeting criteria for either mildly symptomatic cCMV disease or moderately to severely symptomatic cCMV disease). Non-cCMV categories included likely postnatally acquired CMV (lacking symptoms of cCMV and with an initial negative CMV DBS, urine, or saliva test result followed by a positive CMV test result, generally obtained after 21 days of age) and undetermined cCMV status (positive CMV DBS test result obtained after 21 days of age, with no valid CMV test result available prior to 21 days).

## Results

### Study Participants

During the 1-year study period, DBS specimens from 208 322 newborns were submitted for routine NBS. Of these, 101 255 newborns (48.6%) were female and 106 157 (51.0%) were male; the mean (SD) age at specimen collection was 3.5 (12.3) days (median, 1.0 days). In total, 245 newborns (0.1%) were opted out of the CMV study by their parent or guardian, with 223 opt-out requests received prior to release of CMV results (data expunged) and 22 received after release of CMV results (data included), resulting in inclusion of 208 099 total newborns screened (Figure). The characteristics of the screened newborns are described in Table 1.

**Figure.**
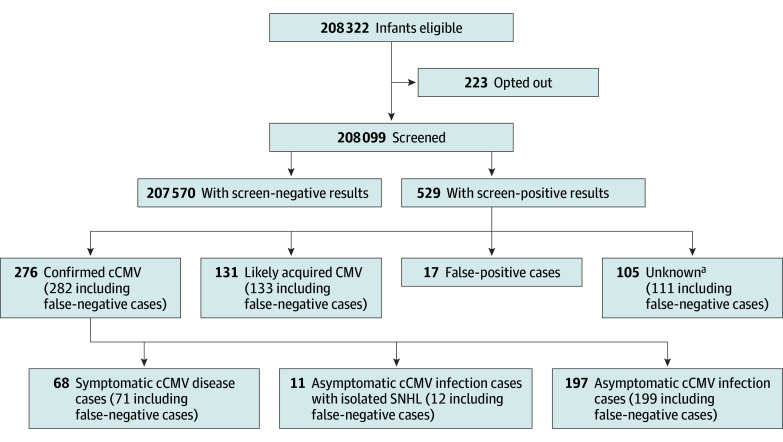
Outcomes of Screening for Congenital Cytomegalovirus (cCMV) SNHL indicates sensorineural hearing loss. ^a^Includes newborns whose specimen was collected past 21 days of age but no suitable prior specimen was available for testing (n = 43), parents declined follow-up (n = 26), or family was lost to follow-up (n = 36).

**Table 1.** Characteristics of Newborns Screened and Referred for Cytomegalovirus

Characteristic	Newborn group, No. (%)^a^	
	Screened	Referred
No. of specimens	247 471	NA
No. of newborns	208 322 (84.2)	529 (100)
No. of newborns opted out by their parents	223 (0.1)	NA
Sex		
Female	101 255 (48.6)	247 (46.7)
Male	106 157 (51.0)	282 (53.3)
Age at specimen collection, d^b^		
≤21	237 411 (95.9)	349 (66.0)
>21	10 059 (4.1)	180 (34.0)
Specimen collection by birth weight		
Low birth weight (<2500 g)	36 262 (14.7)	NA
Normal birth weight (≥2500 g)	211 144 (85.3)	NA
Birth weight		
Low (<2500 g)	18 175 (8.7)	143 (27.0)
Normal (≥2500 g)	190 090 (91.3)	382 (72.2)
NICU stay		
Yes	21 478 (10.3)	178 (33.6)
No	186 844 (89.7)	351 (66.4)
Specimen quantity not sufficient (not tested)^c^	193 (0.08)	NA
Specimen quality unsuitable (tested)^c^	4840 (2.0)	NA

Abbreviations: NA, not applicable; NICU, neonatal intensive care unit.

^a^ Some groups do not sum to the total specimens or newborns due to missing data.

^b^ The mean (SD) age for the entire cohort was 3.5 (12.3 days); the median age was 1.0 days.

^c^ A repeat specimen was requested for these newborns.

### Screen-Positive Results

Positive DBS results were found in 529 newborns (0.3%) who were subsequently referred for clinical evaluation (Table 1 and Figure). Of these, 276 newborns (52.2% of referrals; rate of 1 in 754 [0.1% of newborns screened]) were confirmed to have cCMV, with 68 (24.6%) having symptomatic cCMV disease, 197 (71.4%) having asymptomatic cCMV infection, and 11 (4.0%) having isolated SNHL (Table 2). A further 131 of the 529 referred newborns (24.8%) had likely acquired CMV postnatally, 62 (11.7%) were lost to follow-up or the parents declined follow-up, and 17 (3.2%) had false-positive results. Additionally, 43 of the referred newborns (8.1%) had undetermined cCMV status because the initial suitable specimen submitted to the NBSP was collected after 21 days of age. It was unclear whether these cases were congenital or acquired.

**Table 2.** Outcomes of CMV Referrals and Newborns With Confirmed CMV

Outcome	Referred newborns, No. (%) (N = 529)	Referred newborns with initial CMV-positive DBS specimen collected, No. (%)		Confirmed or potential CMV cases, No. (%) (n = 526)^a^	Incidence ratio (n = 208 099) (1:396)
		At ≤21 d of age (n = 349)	At >21 d of age (n = 180)		
cCMV cases	276 (52.2)	273 (78.2)	3 (1.7)	282 (53.6)	1:738
Symptomatic cCMV disease	68 (12.9)^b^	68 (19.5)^b^	0	71 (13.5)	1:2931
Asymptomatic cCMV infection	197 (37.2)	196 (56.2)	1 (0.6)^c^	199 (37.8)	1:1046
Asymptomatic cCMV infection with isolated SNHL	11 (2.1)	9 (2.6)	2 (1.1)	12 (2.3)	1:17 342
Likely postnatally acquired CMV	131 (24.8)^d^	9 (2.6)^e^	122 (67.8)^d^	133 (25.3)	1:1565
Specimen collected at >21 d of age with no suitable prior specimen available for testing	43 (8.1)	1 (0.3)^f^	42 (23.3)	43 (8.2)	1:4840
Parents declined follow-up	26 (4.9)	18 (5.2)	8 (4.4)	26 (4.9)	1 in 20 referred cases
Lost to follow-up	36 (6.8)	32 (9.2)	4 (2.2)	42 (8.0)^g^	1 in 15 referred cases
False-positive result	17 (3.2)	16 (4.6)	1 (0.6)	NA	1 in 31 referred cases

Abbreviations: cCMV, congenital cytomegalovirus; CMV, cytomegalovirus; DBS, dried blood spot; NA, not applicable; SNHL, sensorineural hearing loss.

^a^ Excludes 17 false-positive cases and includes 14 false-negative cases.

^b^ Includes 1 deceased newborn who likely died of cCMV disease.

^c^ The newborn had a positive urine polymerase chain reaction result at 2 days of age but the initial CMV positive DBS was collected at 28 days of age.

^d^ Includes 2 newborns whose death was not directly attributed to CMV infection.

^e^ All 9 newborns had prior DBS or saliva specimens with negative test results for CMV.

^f^ Specimen was collected at 20 days and the case was considered undetermined cCMV status.

^g^ Includes 4 cases with unknown outcome.

### False-Positive and False-Negative Screen Results

The NBSP was informed by clinicians of 17 newborns with false-positive cCMV screening results (false-positive rate of 0.01%) (Table 3). In 3 cases, the blank spots of each DBS card tested positive for CMV. In 1 case, the false-positive specimen was in close contact to a specimen from a newborn with confirmed CMV and a high viral load born at the same hospital. In 5 false-positive cases, retesting of the specimen confirmed the initial positive result, and in the remaining 8 cases, the CMV NBS PCR results were inconsistent. In these cases, contamination of the NBS card or laboratory contamination could not be ruled out.

**Table 3.** False-Positive and False-Negative CMV Cases

Result	Description	Outcome
No. of false-positive cases		
4	NBS card was contaminated	NA
8	<50% of qPCR results of DBS were positive (possible qPCR contamination)	NA
5	≥50% of qPCR results of DBS were positive (possible NBS card, or qPCR contamination)	NA
No. of initially false-negative cases		
11^a^	Initial DBS qPCR result was negative, but newborn was referred	2 Symptomatic cCMV disease; 6 asymptomatic cCMV infection; 2 likely postnatally acquired; 1 lost to follow-up
5	Initial DBS qPCR result was negative; newborn was not referred; qPCR retest result from a new DNA extraction from the same DBS card was positive	2 Symptomatic cCMV disease; 1 asymptomatic cCMV infection; 1 lost to follow-up; 1 unknown
9	Initial and retest DBS qPCR results were negative; newborn was not referred	1 Symptomatic cCMV disease; 1 asymptomatic cCMV infection; 1 asymptomatic cCMV infection with isolated SNHL; 2 likely postnatally acquired; 1 lost to follow-up; 3 unknown

Abbreviations: DBS, dried blood spot; NA, not applicable; NBS, newborn screening; qPCR, quantitative polymerase chain reaction; SNHL, sensorineural hearing loss.

^a^ Not classified as false negative because a repeat screen using a new DBS punch from specimens from each of these 11 newborns returned a positive result and the newborns were referred for follow-up.

The NBSP was aware of 25 false-negative results (Table 3). In 11 cases, initial DBS PCR results were negative, but the newborns were referred because repeat testing of the DBS from the same card was positive. Repeat testing was performed if discordant results were obtained between twins, or between initial and repeat specimens, or during quality assurance processes. Fourteen newborns were reported as having screen-negative results for CMV but either had clinical features suggestive of cCMV and subsequently tested positive by urine or saliva CMV PCR or had additional CMV testing performed at birth per the institutional NICU protocol. When the NBSP was notified of the false-negative results, the 14 specimens were retested using a new DBS punch, and CMV was detected in 5 cases (Table 3).

### Final Screen-Positive Results

When accounting for 17 false-positive cases and 14 known false-negative cases (Table 3), a total of 526 confirmed CMV cases were identified (0.3% of all newborns screened). Of the confirmed screen-positive and false-negative cases, 282 had cCMV and 133 had postnatally acquired CMV. Thus, the incidence of cCMV was 0.1% (1 in 738) in our screened population (Table 2).

### cCMV Clinical Features

Sixty-eight referred newborns (71 including those with false-negative NBS results) had clinical features of symptomatic cCMV disease at birth (Table 2), including an early-term newborn 1 week of age who likely died of severe cCMV disease. The most common findings included abnormal neuroimaging results (n = 46), microcephaly (n = 10), thrombocytopenia (n = 10), and neutropenia (n = 10) (Table 4). Of the 46 newborns with abnormal neuroimaging results, 26 did not have any any other apparent signs or symptoms. Distinct from newborns with symptomatic cCMV disease at birth, 11 newborns had isolated SNHL but were otherwise considered asymptomatic. Combining 9 symptomatic newborns with SNHL and 12 newborns with isolated SNHL (including 1 with false-negative NBS findings), an estimated overall rate of CMV-related SNHL was 1 in 9909 (0.01%) in this population. Forty-eight of 68 symptomatic newborns (70.6%) and 4 of the 11 newborns with isolated SNHL (36.4%) were treated with valganciclovir hydrochloride.

**Table 4.** Most Commonly Reported Symptoms for Newborns With cCMV Disease

Sign or symptom	All newborns (n = 68)	Newborns with cCMV disease, % (n = 68)	Newborns with cCMV, % (n = 279)
Abnormal neuroimaging	46^a^	67.6	16.5
Microcephaly	10	14.7	3.6
Thrombocytopenia	10	14.7	3.6
Neutropenia	10	14.7	3.6
Sensorineural hearing loss	9	13.2	7.2^b^
Jaundice	8	11.8	2.9
Hepatosplenomegaly	6	8.8	2.2
Petechiae	2	2.9	0.7
Other (eg, small for gestational age, intrauterine growth restriction, elevated liver enzyme levels, hepatitis)	25	36.8	9.0

Abbreviation: cCMV, congenital cytomegalovirus.

^a^ Twenty-four of 46 newborns were determined to have symptomatic cCMV disease based only on imaging studies.

^b^ Includes 9 newborns with symptomatic cCMV disease and 11 with asymptomatic cCMV infection and isolated sensorineural hearing loss.

The largest category of confirmed cCMV cases were asymptomatic, including newborns whose diagnostic workup had been completed (159 of 276 [57.6%]) or those whose diagnostic workup, such as imaging and diagnostic audiology testing, had not been completed (38 of 276 [13.8%]). Inclusive of 6 false-negative cases (Table 3), the ratio of symptomatic cCMV disease (including the newborns with isolated SNHL at birth) to asymptomatic cCMV infection was 83:199 (1:2.4).

Referral center clinicians reported that 39 of 68 symptomatic newborns (57.4%), including the newborn who died of symptomatic cCMV disease, were not recognized as having cCMV until after the positive NBS result. Antiviral treatment was initiated in 25 of these 39 newborns.

### Timing of Screening and Postnatally Acquired CMV

Most referred newborns (349 [66.0%]) had a specimen collected at 21 days or younger, the cutoff age used to define cCMV^19^ (Table 2). In the remainder (180 [34.0%]), the initial positive DBS was collected after 21 days of age. Newborns in the latter group could have congenital or postnatally acquired CMV. None of these 180 newborns had symptomatic cCMV disease, although 2 cases had isolated SNHL. Most newborns referred based on a positive DBS specimen collected after 21 days of age (122 of 180 [67.8%]) were classified as having likely acquired CMV postnatally (Table 2). Additionally, 9 newborns whose initial DBS positive specimen was collected at 21 days or younger were also considered to have acquired CMV. Of the total 131 acquired cases, 128 had between 1 and 4 prior DBS or saliva specimens that tested negative for CMV. For various reasons, 178 of 529 newborns with CMV-positive DBS results (33.6%), including 95 of 131 with postnatally acquired CMV (72.5%), required care in the NICU (eTable 1 in Supplement 1).

## Discussion

In this diagnostic study, we evaluated universal cCMV screening in New York, demonstrating feasibility across a large, geographically and demographically diverse region. Of the 208 322 newborns potentially screened for CMV, only 0.1% of families opted out, indicating high acceptability. This was the first time our program used an opt-out model for a pilot study. Opt-in models are labor intensive and expensive and may prevent some families from benefiting from additional screening for their newborn.

When including cCMV cases with false-negative screen results, the overall cCMV incidence was 1 in 738 (0.1%). Based on prior studies demonstrating incomplete sensitivity of CMV DBS PCR, this is likely an underestimate of true cCMV incidence.^17,22^ To determine assay sensitivity, DBS CMV PCR results should be compared with those of urine PCR, the gold standard diagnostic test. Screening alternative specimen types (ie, urine and saliva) would be logistically challenging for NBS programs. By DBS screen only, our cCMV detection rate of 0.1% is lower than the reported 0.48% in other high-income countries and 0.31% detected in blood or plasma specimens.^1^ NBS has a low tolerance for missing cases, making the high number of false-negative results with CMV screening undesirable. Clearly, diagnostic testing should be performed for newborns at high risk of CMV despite a negative NBS result.

The cCMV screening program in Ontario reported similar logistical challenges and epidemiologic findings to our program.^21^ Both studies were performed across large North American territories. Remarkably, the same low cCMV rate (0.1%) was identified in both screening programs. In contrast, however, the Ontario study identified symptomatic cCMV in 16.0% of newborns, whereas we identified a higher rate of 24.6%. The reason for the difference is unclear. Both programs used similar definitions of symptomatic and asymptomatic cCMV, though the Ontario study used more specific definitions for abnormal laboratory results. Children in Ontario were only referred for pediatric infectious disease consultation if the finding of the initial assessment, performed by a primary care clinician, was abnormal, whereas in New York, referred children were evaluated by pediatric infectious diseases. The studies used different CMV detection assays, which may have different testing characteristics. Finally, differences in populations and local seroprevalence rates may have led to different rates of primary vs repeat maternal CMV infections, resulting in different rates of symptomatic cCMV. These differences underscore the critical need to study cCMV universal screening across varied geographic and demographic settings.

Although newborns with cCMV disease may be identified clinically despite a false-negative CMV newborn screen result, those who have asymptomatic cCMV infection at birth and thus require monitoring may not be. We are aware of 14 newborns with a false-negative CMV screen result who were diagnosed clinically with cCMV or had CMV screening performed (due to the hospital’s NICU protocol) (Table 3). False-negative screen results were expected in this study because of many previous reports of lower sensitivity when DBSs were used as the sample type for detection of CMV.^17,22,23,24,25^ Viral DNA levels are lower in peripheral neonatal blood samples than in urine or saliva and may depend on time of sampling.^26,27,28^ Initial testing of DBS in our study led to 25 false-negative results. In 16 of those cases, retests provided a positive result indicating variation in DBS viremia that could be caused by factors such as uneven distribution of virus in blood and low blood volume.^29^ In 9 false-negative cases, the subsequent NBS results remained negative, probably indicating low levels of CMV viremia at birth.

Forty-eight newborns with symptomatic cCMV disease (70.6%) and 4 with isolated SNHL (36.4%) were treated using valganciclovir. Treatment for cCMV is recommended by the American Academy of Pediatrics for newborns with moderately to severely symptomatic cCMV disease and may be considered for newborns with isolated SNHL.^30^ However, there are insufficient data to recommend therapy for those with mild or asymptomatic cCMV.

Forty-six newborns with cCMV disease were reported to have abnormal neuroimaging findings. Twenty-four of the 46 cases were determined to have symptomatic cCMV disease based only on imaging studies. However, it is not known whether abnormal findings such as subependymal cysts are clinically significant and related to CMV infection.^31^ Parents of newborns evaluated by the referral centers were offered enrollment in a Eunice Kennedy Shriver National Institute of Child Health and Human Development–funded long-term follow-up study, PROACTIVE NYS,^32^ which will study audiological, neurodevelopmental, and quality of life outcomes of children with cCMV and their families.

We identified 131 newborns with likely postnatally acquired CMV. Most of these newborns were admitted to the NICU, an expected finding given the NBSP requirement to repeat DBS submissions in this population. Postnatal infection in healthy term newborns is generally benign. However, in preterm and immunocompromised newborns, infection can cause severe disease. Identification of newborns with postnatally acquired CMV represents another challenge for universal cCMV screening, as they may undergo additional testing and medicalization despite having a much lower risk of long-term complications of CMV than those with congenital infections.

### Limitations

Our study was limited by the lack of a criterion standard for determining the true incidence of cCMV infection and disease. This was demonstrated by the identification of newborns with false-positive and false-negative CMV DBS screen results. We were also unable to characterize newborns with a positive CMV DBS screen who were lost to follow-up, whose parents declined follow-up, or whose first DBS screen was obtained after 21 days of age. cCMV severity grading was limited by our decentralized diagnostic approach, in which clinical findings were summarized and categorized by treating clinicians, rather than having raw clinical data submitted for more uniform disease categorization. The agreed use of the criteria of Rawlinson et al^18^ for grading disease severity by clinicians reduces but does not eliminate interclinician variability. Additionally, although clinicians frequently reported that symptomatic newborns were not recognized until after receiving a positive cCMV screen, the knowledge of having a positive screen may have heightened scrutiny and increased identification of minor abnormalities. While these factors limit interpretation of our findings, they also reflect the clinical challenges^33^ of implementing universal DBS screening for cCMV.

## Conclusions

In this diagnostic study, we demonstrated the feasibility of conducting universal cCMV screening in a large, geographically and demographically diverse US state. The cCMV incidence in New York was lower than most published rates, although true incidence remains unknown due to limited sensitivity of DBS screening. During the 1-year study period, cCMV was diagnosed in 276 newborns, including 68 with symptomatic disease, allowing for timely antiviral initiation when indicated, referral to early intervention programs, and close monitoring for late-onset hearing and neurodevelopmental deficits. More than one-half of newborns with symptomatic cCMV were not recognized prior to the positive NBS result, suggesting that symptom-based cCMV screening may be inadequate for identifying newborns with symptomatic cCMV disease. Long-term data are needed to better understand the impact of cCMV identification among newborns with asymptomatic infection, including a focus on the experiences and perspectives of affected children and their families.

## Notes

Supplement 1. **eMethods.** CMV Screening **eTable.** Outcomes of NICU Newborns Referred and Confirmed with CMV

Supplement 2.
Data Sharing Statement
