# Supplementary material for: Universal Newborn Screening for Congenital Cytomegalovirus Using Dried Blood Spot Specimens
Source: JAMA Netw Open. 2026 Jan 29;9(1):e2554518. doi: 10.1001/jamanetworkopen.2025.54518 (PMC12856680; doi:10.1001/jamanetworkopen.2025.54518)
Supplement: Supplement 1. — eMethods. CMV Screening eTable. Outcomes of NICU Newborns Referred and Confirmed with CMV [file jamanetwopen-e2554518-s001.pdf]

Supplementary Online Content

Tavakoli NP, Sack V, Handel AS, et al. Universal newborn screening for congenital cytomegalovirus using dried blood spot specimens. *JAMA Netw Open.* 2026;9(1):e2554518. doi:10.1001/jamanetworkopen.2025.54518

eMethods. CMV Screening
eTable. Outcomes of NICU Newborns Referred and Confirmed with CMV

This supplementary material has been provided by the authors to give readers additional information about their work.

© 2026 Tavakoli NP et al. *JAMA Network Open*.

eMethods. CMV Screening

For CMV screening, nucleic acid was extracted from two 3.2 mm dried blood spot (DBS) punches using the Extracta™ DBS reagent (Quantabio, Beverly, MA) in a 96-well plate per the manufacturer's instructions with one modification: after incubation at 95°C, plates were centrifuged for an additional 20 min at 3,000 rpm to clear the supernatant. Every 96-well plate included one 3.2 mm punch from each NeoMDx cCMV Kit control DBS (C1, C2 and C3; Revvity, Waltham, MA) as well as a no template control (NTC). Following extraction, nucleic acid amplification and detection was achieved using the NeoMDx™ cCMV PCR Reagent Kit (Revvity), a real-time qPCR assay that amplifies the UL122 region of CMV and the internal control [Ribonuclease P/MRP subunit P30 (RPP30)]. PCR was performed per manufacturer's instructions using the Eonis™ Q384 qPCR instrument (Revvity).

Specimens with an RPP30 PCR result that was out of range [Cycle threshold (Ct) ≥ 30] were re-extracted and re-amplified. Specimens with an acceptable RPP30 PCR result (Ct<30) and no CMV amplification (Ct>40) were reported as screen negative for CMV. Specimens with a positive CMV qPCR result (Ct ≤ 40) were re-extracted in duplicate and qPCR was performed in duplicate on each extract. In addition, qPCR was again performed on the original extract. If ≥3 qPCR results from the 6 total PCR results were positive, the baby was referred for cCMV evaluation.

© 2026 Tavakoli NP et al. *JAMA Network Open*.

eTable. Outcomes of NICU Newborns Referred and Confirmed With CMV

	Number of NICU newborns referred (%)	Number of referred NICU newborns whose initial CMV positive DBS specimen was collected at ≤21 days of age (%)	Number of referred NICU newborns whose initial CMV positive DBS specimen was collected at >21 days of age (%)	Age of collection (days)		
				Range[a]	Average	SD
CMV cases	178	78	100	0-192	30	37.6
Total cCMV cases	65 (36.5)	62 (79.5)	3 (3.0)	0-78	3	10.5
Symptomatic cCMV disease	19[b] (10.7)	19[b] (24.4)	0 (0)	0-3	1	0.6
Asymptomatic cCMV infection	44 (24.7)	43 (55.1)	1 (1.0)	0-28	2	4.2
Asymptomatic cCMV infection with isolated SNHL	2 (1.1)	0 (0)	2 (2)	28-78	53	35.1
Likely postnatally acquired CMV	95[c] (53.4)	7[d] (9.0)	88[c] (88)	3-192	46	31.8
Specimen was collected at >21 days of age but no suitable prior specimen was available for testing	3 (1.7)	0 (0)	3 (3)	127-190	165	33.3
Parents declined follow-up	4 (2.2)	0 (0)	4 (4)	22-149	65	59.5
Lost to follow-up	7 (3.9)	6 (7.7)	1 (1)	0-28	6	10.2
False-positive	4 (2.2)	3 (3.8)	1 (1)	0-30	13	12.3

Abbreviations: cCMV, congenital cytomegalovirus; CMV, cytomegalovirus; DBS, dried blood spot; NICU, neonatal intensive care unit; PCR, polymerase chain reaction; SD, standard deviation; SNHL, sensorineural hearing loss.

[a]Age of collection of zero (0) represents specimens collected at less than 2 hours of age.

[b]Includes one deceased newborn who likely succumbed to cCMV disease.

[c]Includes two deceased newborns whose death was not directly attributed to CMV infection.

[d]All seven newborns had one or more negative DBS or saliva PCR results prior to the DBS specimen that tested positive.

© 2026 Tavakoli NP et al. *JAMA Network Open*.
