# Supplementary material for: Universal Newborn Screening for Congenital Cytomegalovirus Using Dried Blood Spot Specimens
Source: JAMA Netw Open. 2026 Jan 29;9(1):e2554518. doi: 10.1001/jamanetworkopen.2025.54518 (PMC12856680; doi:10.1001/jamanetworkopen.2025.54518)
Supplement: Supplement 2. — Data Sharing Statement [file jamanetwopen-e2554518-s002.pdf]

Data Sharing Statement

Tavakoli. Universal Newborn Screening for Congenital Cytomegalovirus Using Dried Blood Spot Specimens. *JAMA Netw Open*. Published January 29, 2026. doi:10.1001/jamanetworkopen.2025.54518

Data
Data available: No

Additional Information
Explanation for why data not available: Summary data will be readily available, however the work presented is programmatic data from a screening program and families have not consented to sharing their newborn's data. Therefore, individual level data will not be shared. The New York State Newborn Screening does not share data from individual newborns.
